# The Genomic Characteristics of ALK Fusion Positive Tumors in Chinese NSCLC Patients

**DOI:** 10.3389/fonc.2020.00726

**Published:** 2020-05-08

**Authors:** Shaokun Liu, Tanxiao Huang, Ming Liu, Wenlong He, YingShen Zhao, Lizhen Yang, Yingjiao Long, Dandan Zong, Huihui Zeng, Yuanyuan Liu, Wenting Liao, Jingxian Duan, Subo Gong, Shifu Chen

**Affiliations:** ^1^Department of Pulmonary and Critical Care Medicine, The Second Xiangya Hospital, Central South University, Changsha, China; ^2^Research Unit of Respiratory Disease, Central South University, Changsha, China; ^3^Diagnosis and Treatment Center of Respiratory Disease, Central South University, Changsha, China; ^4^HaploX Biotechnology Co., Ltd., Shenzhen, China; ^5^Department of Geriatrics, The Second Xiangya Hospital, Central South University, Changsha, China

**Keywords:** NSCLC, ALK, NGS—next generation sequencing, copy number aberrations, genomic landscape

## Abstract

Anaplastic lymphoma kinase (ALK) fusion events account for ~3–7% genetic alterations in patients with non-small cell lung cancer (NSCLC). In this study, we identified the ALK fusion patterns and a novel ALK fusion partner in 44 ALK positive NSCLC patients using a customized HapOncoCDx panel, and identified ALK fusion partners. The most common partner is EML4, forming the variant 1 (v1, E13:A20, 18/44), variant 2 (v2, E20:A20, 5/44), and variant 3 (v3, E6:A20, 13/44). Moreover, we detected a new ALK fusion partner HMBOX1. At the mutation level, TP53 is the most frequently mutated gene (24%), followed by ALK (12%) and STED2 (12%). The median tumor mutation burden (TMB) of these samples is 2.29 mutations/Mb, ranging from 0.76 mut/Mb to 16.79 muts/Mb. We further elaborately portrayed the TP53 mutation sites on the peptide sequence of the encoded protein by lollipop. The mutational signature and copy number alterations (CNAs) of the samples were also analyzed. The CNA events were found in 13 (13/44) patients, and the most commonly amplified genes were MDM2 (*n* = 4/13) and TERT (*n* = 4/13). Together, these results may guide personalized clinical management of patients with ALK fusion in the era of precision medicine.

## Introduction

Anaplastic lymphoma kinase (ALK) fusion events, which are the result of ALK rearrangements, account for ~3–7% genetic alterations in non-small cell lung cancer (NSCLC) patients (1, 2). These oncogenic mutations could lead to the constitutive activation of the ALK tyrosine kinase domain, and further cause tumorigenesis (3). Hitherto, multiple ALK fusion partners have been identified, and the most normal one is echinoderm microtubule-associated protein-like 4 (EML4), which were observed in nearly 80% of all the ALK fusion cases (2). It is worth noting that more than a dozen of different EML4-ALK variants have been identified in NSCLC patients. The most common variants are variant 1 (v1, E13:A20), variant 2 (v2, E20:A20), and variant 3 (v3, E6:A20) (4).

Currently, ALK tyrosine kinase inhibitors (TKIs) are recommended for the treatment of NSCLC patients harboring ALK fusion (5). Prior to ALK TKIs treatment, the median overall survival (OS) of ALK fusion-positive metastatic NSCLC patients receiving chemotherapy was around 12 months (6). However, under the sequential treatment with ALK fusion TKIs, the OS of the patients were extended to 5 years (7). The first approved is Crizotinib which is the first generation TKI. Compared to traditional chemotherapy, it improves the PFS and OS of ALK fusion NSCLC patients significantly (8). Nevertheless, nearly all of the patients would develop drug resistance within 2 years after the initial treatment. The drug resistance was possibly caused by a secondary mutation in the kinase domain of ALK, the activation of alternative pathways, ALK amplification, or epithelial-mesenchymal transition (9). To overcome the resistance, the second-generation ALK TKIs were developed including Ceritinib, Alectinib, and Brigatinib. They were approved for the treatment of metastatic NSCLC patients with ALK fusion and had progressed on or intolerant to Crizotinib. Notably, Ceritinib and Alectinib were approved for the first-line treatment of the ALK fusion positive metastatic NSCLC patients (10–16). In addition, as the third generation ALK inhibitor, Lorlatinib has also been approved for the treatment of metastatic NSCLC patients with ALK fusion, on condition that the disease has progressed on Crizotinib or at least one other ALK inhibitor such as Alectinib or Ceritinib for metastatic disease (17). It is worth noting that different ALK inhibitors have different potencies and spectrums against acquired resistance mutations (18).

In the era of precision medicine, the genomic profiles of the patients may guide the planning of treatment strategy. For the ALK fusion positive NSCLC patients, detailed genomic profiles can elucidate the fusion partner and the rearranged breakpoint. Moreover, the proposed resistant mutations are critical for clinical treatment guidelines. Furthermore, several studies reported that molecular profiling is also associated with the prognosis of patients. Christopoulos et al. reported that the concomitant TP53 mutation is a strong indicator of poor prognosis in ALK fusion positive NSCLC patients (19). Their study also reported that EML4-ALK fusion variant V3 was associated with a more aggressive phenotype and worse overall survival due to earlier failure of several therapeutic modalities. In addition, they found that V3 and TP53 double positive patients had a very high risk of death with a median OS of around 2 years.

With the development of next-generation sequencing technologies, it is becoming more affordable to obtain the genomic landscape of cancer patients. In this study, we aim to demonstrate the genomic landscape of ALK fusion-positive tumors in 44 Chinese NSCLC patients sequenced with our customized HapOncoCDx panel which involves hybridization capture and deep sequencing of all protein-coding exons of 464 cancer-associated genes and other selected introns of other oncogenes and tumor suppressor genes, and illustrate their genomic mutation patterns and characteristics, which potentially helps to develop treatment strategy.

## Materials and Methods

### Patients and Samples

Forty-four patients were enrolled from 1349 NSCLC patients in this study. Tumor tissues were collected during surgery, and were formalin fixed, paraffin-embedded (FFPE) and archived. Peripheral blood (PBL) samples were also collected from each patient as control.

### DNA Extraction

DNA samples were extracted from Formalin-fixed paraffin-embedded (FFPE) samples with QIAamp DNA FFPE tissue kit (Qiagen). Extraction of PBL DNA was conducted using the RelaxGene Blood DNA system (Tiangen Biotech Co., Ltd., Beijing, China) according to the manufacturer's protocol. All the DNA samples were quantified both using the Qubit 2.0 fluorometer and the Qubit dsDNA HS Assay kit (Thermo Fisher Scientific, Inc., Waltham, MA, USA) according to the manufacturer's protocol.

### Library Construction and Sequencing

One hundred nanogram of DNA from each sample was sheared by the dsDNA Fragmentase (New England BioLabs, Inc., Ipswich, MA, USA), and then performed size selection (150–250 bp) using Ampure XP beads (Beckman Coulter, Inc., Brea, CA, USA). Library construction was performed using the KAPA Library Preparation kit (Kapa Biosystems, Inc., Wilmington, MA, USA) according to the manufacturer's protocol. The concentration of the library were assessed using the e Qubit dsDNA HS Assay kit, and fragment length was determined on a 4200 Bioanalyzer (Agilent Technologies, Inc., Santa Clara, CA, USA). Target enrichment was carried out using the Agilent SureSelect XT HS kit (Agilent Technologies) according to the manufacturer's Protocol. DNA sequencing was then performed on the Illumina Novaseq 6000 system at an average depth of 2000X.

### Data Analysis and Variant Calling

Raw sequences were pre-processed by fastp version 0.18.0 (https://github.com/OpenGene/fastp) (20), and clean reads were aligned to the hg19 genome (GRch37) using Burrows-Wheeler Aligner maximal exact matches algorithm (21). The Gencore version 0.12.0 (https://github.com/OpenGene/gencore) (22) was used for removing duplicate reads. Pileup files with mapping quality ≥60 were generated using Samtools version 0.1.19 (http://www.htslib.org/) (23). Somatic variants were called using VarScan2 version 2.3.8 (http://varscan.sourceforge.net/) (24) [the minimum read depth 20; the variant allele frequency (VAF) threshold ≥0.01; somatic-*P* ≤ 0.01; strand-filter ≥1; others, default parameters]. CNV kit with version 0.9.3 (https://github.com/etal/cnvkit) (25) was used for copy number variation detection, and GeneFuse version v0.6.1 (https://github.com/OpenGene/GeneFuse) (26) for structural variation detection. Maftools was used for visualizing somatic variant analysis (27).

## Results

### Sample Collection and Patient Characteristics

Of the 1349 NSCLC cases, ALK rearrangements were detected in 44 cases (3.26 %). Those 44 Chinese patients with locally advanced or metastatic NSCLC were enrolled in this study, of which 20 (45.5%) were female. All patients carry ALK fusion events. Their mean age was 52.5 with ranging from 29 to 73. NGS was performed on 44 pairs of tumor and white blood cell samples. All the samples that passed the histology quality control (HQC) yielded sufficient amounts of DNA for NGS.

### Identification of ALK Rearrangements Using Targeted Sequencing

**Figure 1:**
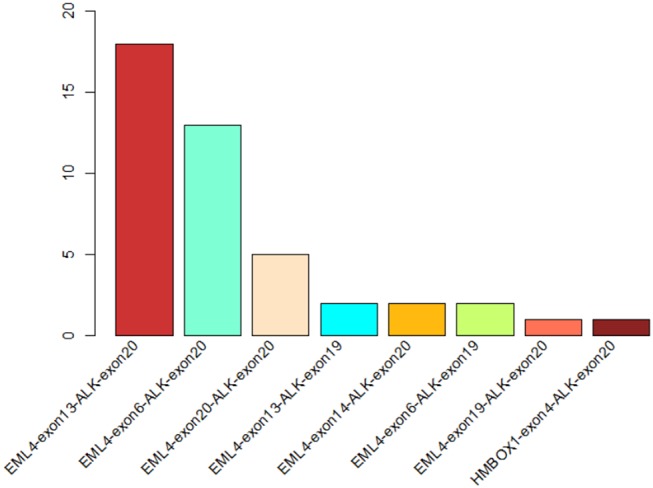
The statistics of different ALK rearrangement forms. The number of each ALK fusion pattern identified in 44 NSCLC patients are shown in the barchart.

**Figure 2:**
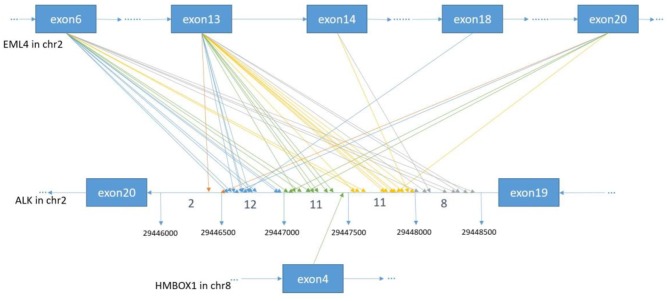
The breakpoint distribution in ALK and the respective fusion partners. Each arrowed line represents one fusion event. The exact breakpoints of ALK in GRch37 are shown in the middle panel, while the fused exons of ALK fusion partners are shown in the top and bottom panels, respectively. The sequence of ALK is exhibited reversely (from right to left), while the sequences of EML4 and HMBOX1 are represented in the forward direction. The genomic region of ALK between 299446000 and 29448500 on Chromosome 2 is divided into regions every 500 bp. Breakpoint position in ALK locate between 29446000 and 29446500 with an orange arrow, between 29446501 and 29447000 with a blue arrow, between 29447001 and 29447500 with a green arrow, between 29447501 and 29448000 with an yellow arrow, between 29448001 and 29448500 with a gray arrow.

In order to identify ALK rearrangement from the DNA of patients' FFPE samples, we designed probes to cover the intron 18 and intron 19 of ALK, as well as introns of some well-known ALK fusion partners. We identified ALK rearrangements and corresponding breakpoints in the sequencing data of these patients. The statistical summary and breakpoints of the rearrangement events are listed in Table 1 and shown in Figures 1, 2, respectively. We found that 43 out of 44 patients had an EML4-ALK fusion, with variant 1 (v1, E13:A20), variant 2 (v2, E20:A20), and variant 3 (v3, E6:A20) detected in 18, 5, and 13 patients, respectively. We also identified one novel ALK fusion partner HMBOX1.

**Table 1:** Fusion patterns of ALK.

**Fusion type**	**Counts**	**Percent (%)**
EML4-exon13-ALK-exon20	18	40.9
EML4-exon6-ALK-exon20	13	29.5
EML4-exon20-ALK-exon20	5	11.4
EML4-exon13-ALK-exon19	2	4.5
EML4-exon14-ALK-exon20	2	4.5
EML4-exon6-ALK-exon19	2	4.5
EML4-exon19-ALK-exon20	1	2.3
HMBOX1-exon4-ALK-exon20	1	2.3

### Mutational Profiles of ALK Fusion Positive NSCLC Patients

**Figure 3:**
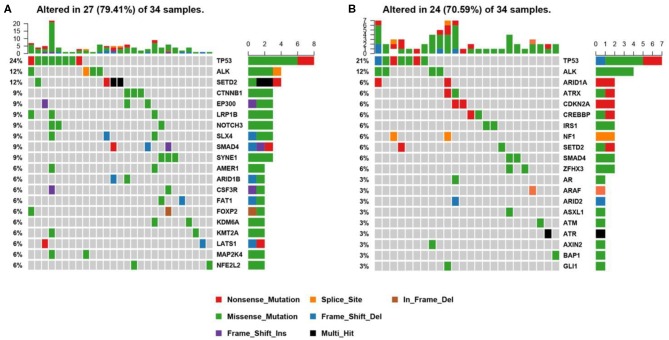
Mutational profiles of ALK positive NSCLC patients. **(A)** This is the oncoprint of the somatic SNVs and Indels in 34 patients in our study. Somatic alterations included missense, nonsense, frameshift indel, in-frame indel, splice site, translation start site, multi_Hit mutations. The genes are ranked by the frequency of the mutations across all samples. **(B)** This is the oncoprint of the somatic SNVs and Indels in 34 patients from the MSK-IMPACT study. Somatic alterations included missense, nonsense, frameshift indel, in-frame indel, and splice-site mutations. The genes are ranked by the frequency of the mutations across all samples.

Genomic alterations were found in 34 (*n* = 34/44, 77.3%) samples with a total of 134 alterations identified including variants of non-synonymous mutations and splicing mutations. The detailed information is shown in Figure 3A. The mutation landscapes of ALK fusion positive NSCLC patients were highly heterogeneous. The median TMB was 2.29 mut/Mb with a range between 0.76 and 16.79 mut/Mb which is similar to the TMB of the TCGA NSCLC cohort.

We constructed a heatmap to demonstrate the somatic mutations occurred in the tumor tissues of the patients (Figure 3A). TP53 was most commonly altered (*n* = 8/34, 24%), followed by SETD2 (*n* = 4, 12%), ALK (*n* = 4, 12%), SYNE1 (*n* = 3, 9%), SMAD4 (*n* = 3, 9%), SLX4 (*n* = 3, 9%), NOTCH3 (*n* = 3, 9%), LRP1B (*n* = 3, 9%), EP300 (*n* = 3, 9%), and CTNNB1 (*n* = 3, 9%).

Other genomic alterations of low frequencies are AMER1 (*n* = 2, 6%), ARID1B (*n* = 2, 6%), CSF3R (*n* = 2, 6%), FAT1 (*n* = 2, 6%), FOXP2 (*n* = 2, 6%), KDM6A (*n* = 2, 6%), KMT2A (*n* = 2, 6%),LATS1 (*n* = 2, 6%), MAP2K4 (*n* = 2, 6%), NFEL2L2 (*n* = 2, 6%), NOTCH1(*n* = 2, 6%), NTRK3 (*n* = 2, 6%), TERT (*n* = 2, 6%), and TGFBR2 (*n* = 2, 6%). Alterations in ABL1, ADH1B, ALDH2, APC, AR, ARID2, ATM, AURKA, BMPR1A, CACNA1C, CADM2, CAMTA1, CAPN2, CARD11, CDC73, CDK12, CREBBP, CSMD3, DNMT3A, EPHA3, ERBB4, ESR2, EWSR1, EXT1, EZH2, FGFR1, FLCN, FOXA1, FOXL2, GATA6, GPRIN2, HIF1A, HNF1B, JAK1, KDR, KMT2C, KMT2D, LATS2, MAP2K1, MDM4, MYCN, NF2, NSD1, NTRK1, PTEN, PTPRD, PZP, RARA, RIT1, RNF43, ROS1, SETBP1, SMARCA4, SMO, SOCS6, SOX2, SPEN, STAT3, STK11, SUZ12, TSHR, and U2AF1 were identified in one sample each (*n* = 1, 3%). We further compared our results with the MSK-IMPACT study (28), in which we extracted 45 ALK fusion positive cases that yielded 81 mutations. Overall, our results were highly consistent with the MSK-IMPACT findings, which showed that TP53 and ALK are the most frequently altered genes (Figure 3B).

**Figure 4:**
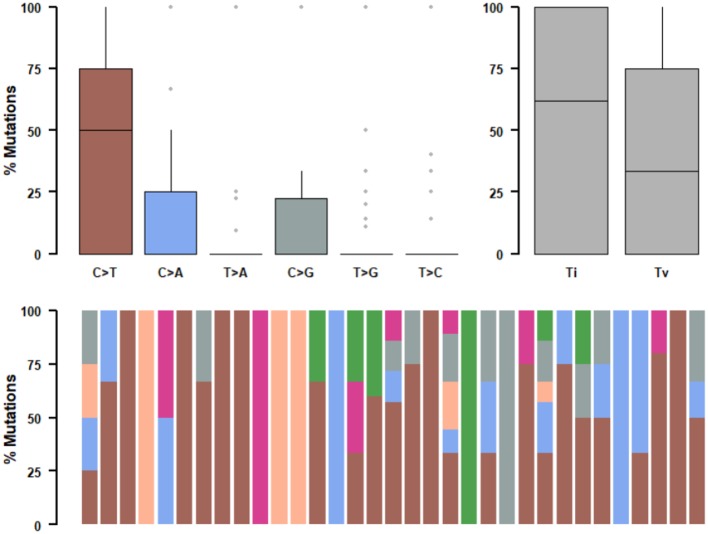
Mutational signatures of ALK fusion positive NSCLC patients. SNPs are classified into transitions and transversions. Summarized data are visualized as a boxplot showing overall distribution of six different conversions **(Top)** and as a stacked barplot showing the fraction of conversions in each sample **(Bottom)**.

We further studied their mutational signatures. We observed that C>T transition occurred most frequently, followed by C>A transition (Figure 4). This pattern is consistent with COSMIC signature 1 that had been found in most cancer samples.

**Figure 5:**
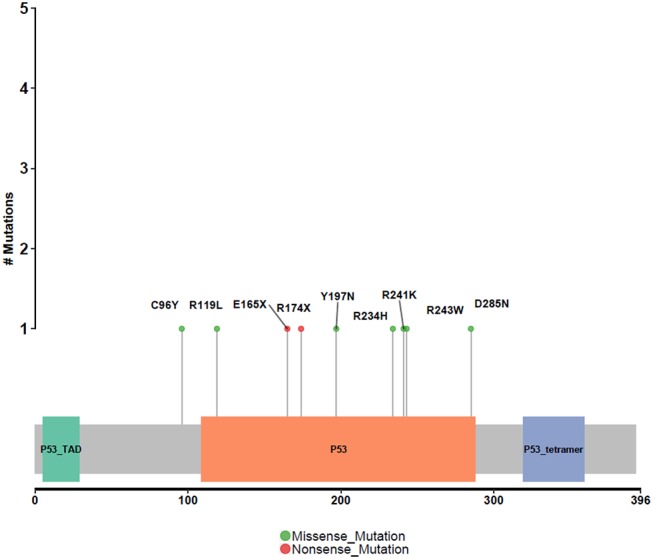
Protein variants resulted from TP53 mutations. Protein variants caused by TP53 mutations are displayed in the lollipop plot. These sites are considered to be mutational hot-spots.

Different driver gene mutations revealed inter-tumor heterogeneity. TP53 mutations in exon 5–8 were observed, and we further elaborately portrayed the TP53 mutation sites on the peptide sequence in a lollipop plot (Figure 5).

### Copy Number Aberrations of ALK Fusion Positive NSCLC Patients

**Figure 6:**
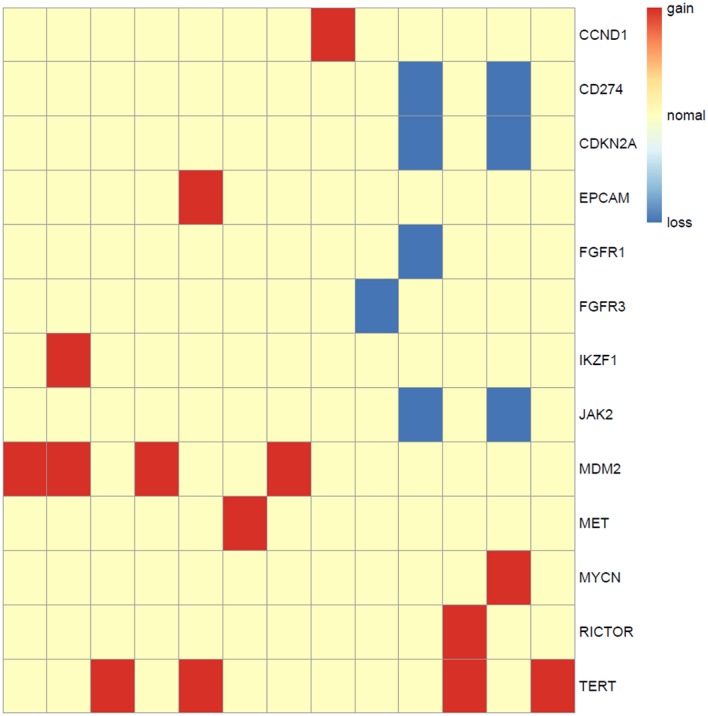
Copy number aberrations in 13 ALK fusion positive NSCLC patients. The names of the aberrant genes are shown in the y-axis, while each column represents a patient. The type of copy number aberrations, including gain, normal, and loss are indicated by red, yellow, and blue, respectively.

Somatic copy number alterations were found in 13 (*n* = 13/44, 29.5%) samples. A total of 22 alterations were identified, including gain and loss (Figure 6). MDM2 and TERT were most commonly amplified genes (*n* = 4/13, 31%), followed by CCND1, EPCAM, IKZF1, MET, MYCN, RICTOR (*n* = 1, 8%). Loss of copy number was most frequently observed in CD274, CDKN2A, JAK2 (*n* = 2/13, 15%), followed by FGFR1, FGFR3 (*n* = 1, 8%).

## Discussion

In this study, we identified ALK rearrangement events in 44 Chinese NSCLC patients using NGS technologies. Consistent with other studies, the most common ALK fusion partner is EML4, and the fusion occurs in the forms of the three most common variants. We also report a novel ALK fusion partner HMBOX1. It implied that NGS-based assessment for ALK fusions was accurate and comprehensive, having the unique advantages in detecting unknown ALK fusion partners, and identifying the exact breakpoints compared to the traditional methods, like FISH and IHC.

At the same time, we characterized the mutational profiles of the patients. The results were consistent with other studies, in terms of the relatively lower frequency of TP53 mutations, lower TMB, and fewer co-mutations compared to ALK-negative NSCLC patients (29). Besides, we identified the copy number alterations in their genome. Apart from the genes with a high frequency of copy number amplification, such as MET, MDM2, and TERT, we also identified some genes with copy number loss, such as CD274, CDKN2A, and JAK2. This information is important for guiding optimal clinical treatment. For instance, the copy number loss of CD274 probably indicates a low expression of PD-L1. MDM2 amplification had been reported to associate with a poor clinical outcome and significantly increased tumor growth rate with anti-PD-1/PD-L1 immunotherapies (30).

In conclusion, using our customized HapOncoCDx panel, we not only successfully detected the ALK fusion events in 44 Chinese NSCLC patients, but also explored their genomic mutational landscapes. To the best of our knowledge, this is the first study that exhibited the mutational landscape of Chinese NSCLC patients with ALK rearrangement. This result can provide genomic information for personalized clinical management for patients with ALK fusion in the era of precision medicine.

## Data Availability Statement

The raw sequence data reported in this paper have been deposited in the Genome Sequence Archive in BIG Data Center, Beijing Institute of Genomics (BIG), Chinese Academy of Sciences, under accession numbers HRA000138, HRA000138 that can be accessed at http://bigd.big.ac.cn/gsa-human.

## Ethics Statement

This study was approved by the ethics committee of the Second XIANGYA Hospital of Central South University and complied with Good Clinical Practices, the principles of the Declaration of Helsinki and all applicable regulatory requirements. All patients provided written informed consent prior to any study-specific procedures.

## Author Contributions

SL, TH, SG, and SC designed the study. WH, YZ, LY, YLo, DZ, HZ, and YLi carried out the sequencing experiment and collected data. TH, YZ, and WL performed the bioinformatics analysis. TH, SL, and ML wrote this manuscript. JD and SC revised this manuscript. SG and SC supervised the study.

## Conflict of Interest

TH, ML, YZ, YLi, WL, JD, and SC were employed by the company HaploX Biotechnology Co., Ltd. The remaining authors declare that the research was conducted in the absence of any commercial or financial relationships that could be construed as a potential conflict of interest.
